# Inpatient Hospitalization Costs: A Comparative Study of Micronesians, Native Hawaiians, Japanese, and Whites in Hawai‘i

**DOI:** 10.3390/ijerph13010029

**Published:** 2015-12-22

**Authors:** Megan Hagiwara, Deborah Taira Juarez, Seiji Yamada, Jill Miyamura, Tetine Sentell

**Affiliations:** 1 Office of Public Health Studies, University of Hawai‘i at Manoa, 1960 East-West Road, Honolulu, HI 96822, USA; 2 Daniel K. Inouye College of Pharmacy, University of Hawai‘i at Hilo, 677 Ala Moana Boulevard, Suite 1025, Honolulu, HI 96813, USA; dtjuarez@hawaii.edu; 3 Department of Family Medicine and Community Health, John A. Burns School of Medicine, University of Hawai‘i, 95-390 Kuahelani Avenue, Mililani, Hawai‘i 96789, USA; seiji@hawaii.edu; 4 Hawaii Health Information Corporation, 733 Bishop St # 1870, Honolulu, HI 96813, USA; jmiyamura@hhic.org; 5 Office of Public Health Studies, University of Hawai‘i, 1960 East-West Road, Honolulu, HI 96822, USA; tsentell@hawaii.edu

**Keywords:** hospitalization, costs, Pacific Islander, Micronesians, health equity

## Abstract

Considerable interest exists in health care costs for the growing Micronesian population in the United States (US) due to their significant health care needs, poor average socioeconomic status, and unique immigration status, which impacts their access to public health care coverage. Using Hawai‘i statewide impatient data from 2010 to 2012 for Micronesians, whites, Japanese, and Native Hawaiians (N = 162,152 hospitalizations), we compared inpatient hospital costs across racial/ethnic groups using multivariable models including age, gender, payer, residence location, and severity of illness (SOI). We also examined total inpatient hospital costs of Micronesians generally and for Medicaid specifically. Costs were estimated using standard cost-to-charge metrics overall and within nine major disease categories determined by All Patient Refined Diagnosis Related Groups. Micronesians had higher unadjusted hospitalization costs overall and specifically within several disease categories (including infectious and heart diseases). Higher SOI in Micronesians explained some, but not all, of these higher costs. The total cost of the 3486 Micronesian hospitalizations in the three-year study period was $58.1 million and 75% was covered by Medicaid; 23% of Native Hawaiian, 3% of Japanese, and 15% of white hospitalizations costs were covered by Medicaid. These findings may be of particular interests to hospitals, Medicaid programs, and policy makers.

## 1. Introduction

The health care costs of Micronesians in the United States (US) is a topic of increasing importance to health care administrators, health disparities researchers, and health policy makers, as Micronesians are a growing population in the US [1] who often have limited financial resources and significant healthcare needs [2,3] coupled with a unique immigration status that affects their access to public health care coverage [4,5].

Micronesia is made up of thousands of islands and atolls in the middle of the Pacific Ocean. The political relationship between Micronesian nations and the US began during World War II. After the war, the US military conducted major nuclear testing in the region, with significant health consequences [6,7]. In the 1980s, three Micronesian nations—the Republic of Palau, the Republic of the Marshall Islands, and the Federated States of Micronesia—signed a series of treaties with the US known as the Compacts of Free Association (COFA). The Compacts gave the US exclusive military control over the region along with the obligation to strengthen the education and health infrastructure. From these agreements, citizens of these Micronesian nations can legally work and live in the US without visas, health clearances, or time limits [4,5]. In legal terms, they are not considered immigrants, and are called “COFA migrants” [8].

Soon after the Compacts were enacted, the number of citizens of COFA nations migrating to the US increased [9]. In the US, from 2000 to 2010 the Chuukese and Marshallese populations (two COFA migrant ethnicities) grew 544% and 237%, respectively [1]. Many COFA migrants arrive in the US with limited experience with the English language, lack of familiarity with the US health care system, and limited financial resources [2,3,4]. Many Micronesians in the US also have serious health needs, including a high burden of diabetes, tuberculosis, and certain cancers [2,10], making access to health care a topic of vital importance to this population [5].

Initially, COFA migrants were eligible for Medicaid under federal guidelines. However, under the federal Personal Responsibility and Work Opportunity Reconciliation Act of 1996 (PRWORA), COFA migrants were deemed ineligible for federal Medicaid coverage. In contrast to immigrants who can become eligible for Medicaid after five years of residence, under PRWORA, COFA migrants can never qualify for Medicaid under federal guidelines regardless of how many years they live in the US.

After PRWORA passed, some states continued to allow Medicaid participation for COFA migrants who were not otherwise insured. The cost of this coverage was subsidized by the states. Hawai‘i, which has the largest proportion of the COFA migrants in the US, originally provided coverage to COFA migrants through its state Medicaid program. In 2009, the Hawai‘i state government, citing economic reasons, started to disqualify COFA migrants from its Medicaid rolls. After a long legal battle, with appeals up to the US Supreme Court, in late 2014, it was deemed legal for the State of Hawai‘i to discontinue Medicaid coverage for COFA migrants. Soon after, the state announced that non-pregnant, non-aged, blind, or disabled (non-ABD) COFA migrant adults were no longer eligible for state-funded Medicaid. Instead, these individuals were expected to enroll in private insurance plans through the state insurance exchange, with premiums for many currently subsidized by the state [11]. Although the total population of COFA migrants is difficult to measure, a recent estimate reported 15,000 COFA migrants resided in Hawai‘i [10]. Other states with significant Micronesian populations, including Arkansas, have made different health policy decisions [12].

Despite considerable policy action with important consequences in health care equity, access, quality, and outcomes for the Micronesians population, little quantitative evidence exists regarding the cost of health care for this population. In fact, limited data exists on the health care utilization of this population generally. Only very general estimates of health care and other social costs exist. For instance, the state of Hawai‘i estimated that in 2007 it spent $101 million in social and health costs for COFA migrants including $37 million by the Department of Human Services [13]. This data was estimated from expenditures by the various departments of the state executive branch, e.g., Department of Human Services, Department of Education, Department of Justice. In November 2014, the state estimated that the annual Medicaid expenditure on COFA migrants was $49 million [14]. In March 2015, the Department of Human Services cited “savings” of approximately $23,000,000 per year from the cessation of Medicaid benefits for identified non-citizens [15].

The current study seeks to address a critical evidence gap regarding health care costs for this population focusing on inpatient hospital costs, a notably expensive part of the US health care system. We examined total inpatient hospital costs for Micronesians generally and for those covered by Medicaid specifically in the state of Hawai‘i. We also compared Micronesian inpatient hospital costs to those of three other major racial/ethnic groups (whites, Japanese, and Native Hawaiians). Whites were included because most often national, race-based health comparison research uses them as the reference group. Japanese were included as they are one of the healthiest groups in Hawai‘i [16,17]. Native Hawaiians were included because they are a larger and better studied Pacific Islander population group than Micronesians and, in Hawai‘i, Native Hawaiians suffer from significant health inequities and have higher health care costs for some types of preventable hospitalizations compared to other major racial/ethnic groups in the state [18,19].

## 2. Experimental Section

### 2.1. Data

Discharge data from all 24 acute care, non-military hospitals operating in Hawai‘i from 2010 to 2012 were obtained from the Hawaii Health Information Corporation (HHIC) inpatient data [20]. HHIC manages, de-identifies, and verifies hospital data for all visits and all payers. Other variables used from this database included patients’ race/ethnicity, age, gender, insurer, and diagnosis from primary and secondary International Classification of Diseases—9th revision—Clinical Modification (ICD-9) diagnostic codes. (The one military hospital in the state does not provide disaggregated race/ethnicity information for Asian or Pacific Islander populations and therefore could not be included in this analysis.) This study was deemed exempt by the University of Hawai‘i Committee on Human Studies.

### 2.2. Sample

Adult (18 years and older) non-pregnancy-related hospital admissions in Hawai‘i from 2010 to 2012 for Micronesians Native Hawaiians, Japanese, and whites were considered (N = 162,401). Pregnancy-related hospitalizations were identified and excluded using Medical Diagnostic Category (MCD) 14 (*pregnancy*, *childbirth, and puerperium*). Discharges missing data on patient’s payer type (n = 1) or location of residence (n = 248) were excluded for a final study sample of 162,152.

### 2.3. Costs

Costs were estimated using established cost/charge ratio (CCR) guidelines available from the Agency of Healthcare Research and Quality [21]. This allows for the conversion of charge data to cost estimates, including an adjustment factor (AF) for differences in case mix (as measured by All Patient Refined Diagnosis Related Groups (APR-DRG) and severity of illness). Relevant data is available to hospitals in states that participate in the Cost-to-Charge Central Distributor, which includes Hawai‘i. Each hospital has a unique cost adjustment factor. To compute estimated cost the following formula was used: (Hospital Charges * CCR) * (*APRDRG Adjustment Factor* (*APRDRG-AF*)) *= Estimated cost.* All costs were adjusted to constant 2012 dollars using the Medical component of the Consumer Price Index [22].

### 2.4. APR-DRGs

Costs were compared in general and specifically within disease categories, which were determined using the All Patient Refined Diagnosis Related Groups (APR-DRGs) from 3M grouper version 29 [23]. APR-DRGs are derived by grouping discharge ICD-9 codes [18] and are used to measure healthcare related resource outcomes. APR-DRGs were designed as an all-payer alternative to Diagnosis Related Groups (DRGs), which were developed to classify hospitalizations for Medicare populations specially and focus only on diagnostic categories of relevance to Medicare [24]. APR-DRGs categorize each hospitalization according to the primary reason for hospitalization. While any secondary reasons for hospitalization or co-morbidity is not represented by the main APR-DRG category, each APR-DRG also has associated severity of illness (SOI) and risk of mortality categories [24]. The presence of co-morbidities would be associated with higher SOI scores. Due to the large number of available APR-DRGs (>300) [23] and the goal of providing a general portrait of hospitalized Micronesians, instead of looking at individual APR-DRGs, we classified them by grouping related APR-DRGs under a limited number of overarching disease categories: cancer, infectious disease, chronic diseases, and behavioral health issues. Appendix Table A1 provides information regarding the APR-DRG groupings. More details about each disease categories, including the number of hospitalizations by APR-DRG categories, are included elsewhere [25].

### 2.5. Analyses Samples

Multiple hospitalizations of the same individual within each disease category were identified using their unique ID number assigned in the database. Only the first entry for each individual was used for racial/ethnic comparisons of costs in unadjusted and adjusted models. This was to prevent individuals who were hospitalized multiple times from skewing racial/ethnic group comparisons. This left a study population of 69,449 unique individuals for the racial/ethnic cost comparisons within APR-DRGs and a study population of 109,258 unique individuals for the “overall” analyses. For the cumulative cost estimates for Micronesians overall and by disease categories, all admissions by individuals were included to identify the full cost burden.

### 2.6. Race/Ethnicity

Race/ethnicity was the main independent variable. The HHIC race/ethnicity variable was collected consistently (with a minor exception noted below) across all included hospitals in Hawai‘i during the study period and is self-reported by patients at intake as one primary race/ethnicity. Mixed race patients are thus included as their primary self-reported racial/ethnic identity. We focused on Micronesians and three comparison populations: Native Hawaiians, Japanese, and whites. The Micronesian category included individuals reporting Marshallese or Other Micronesian (excluding Guamanian and Chamorro) as their primary racial/ethnic identity. The Micronesian category likely includes COFA migrants, immigrants from other Micronesian nations, and Micronesians born in the US or in the US-affiliated Pacific.

Data from two small, rural hospitals did not conform to the race/ethnicity specifications needed to identify all Micronesian groups during the year of 2010. The impact on study results was likely minimal as the combined number of Micronesian hospitalization from these two hospitals during 2010 was 18 total and 23 total Micronesian hospitalizations per year for 2011 and 2012, respectively.

### 2.7. Insurance Status

Insurance status was categorized as Department of Defense (insurance available to active military and their families), Medicare, Medicaid, Private, or Self Pay. For the total cost analyses, we focus on the Medicaid *vs.* other payment type due to the policy relevance of Medicaid costs for the Micronesian population specifically.

### 2.8. Control Variables

Severity of illness was determined using 3M classification methods within the APR-DRG [23]. The 3M severity-of-illness classification method considers several pieces of information including primary and secondary diagnoses, procedures from ICD-9-CM discharge codes, as well as the patient’s age, sex, and discharge disposition. SOI is scored on a scale of 1 to 4, with higher scores indicating greater severity.

For most hospitalizations, coverage type is highly associated with costs. Therefore, in our multivariable model we controlled for type of insurance as well as age, gender, insurance status, and location of residence. Age was measured continuously 0–90 years (top-coded). All persons 90 years and above were reported as 90 in the data set to maintain confidentially. Gender was female or male. Location of residence was categorized as Hawai‘i not Oahu, Oahu, and Outside Hawai‘i. Location of patient’s residence was included to capture possible differences caused by limited availability of healthcare on Hawai‘i’s neighbor islands, including primary, mental health, and specialized care [26].

### 2.9. Statistical Analysis

STATA 12.0 [27] was used to conduct the analysis. To describe the total cost burden for Micronesians, we summed the total costs of all hospitalizations overall and then within disease type for Micronesians over the three year period. We considered these costs by Medicaid *vs.* not Medicaid.

Costs by hospitalization were compared by racial/ethnic group using Kruskal-Wallis tests due to the highly skewed distribution of cost data. Costs by race/ethnicity adjusting for other factors were then predicted in a multivariable model using the gamma regression with log link [28]. Gamma regression with log link is commonly used in cost data analyses and has been found to particularly suitable to address the potential for highly skewed distribution in cost data [29].

The first set of multivariable models included age, gender, insurance status, and location of residence. The second set of multivariable models included age, gender, insurance status, location of residence, and severity of illness (SOI) to specifically investigate the role of severity of illness in explaining differences in costs by race/ethnicity. These variables were chosen a priori based on previous research and theory as likely to be significant in predicting costs [25]. Thus, all the models included the same set of control variables.

Multivariable models were used to calculate cost ratio estimates (RE) and 95% confidence intervals (CI) for each racial/ethnic group compared with Micronesians (our focal population) after adjustment for other factors. A two-tailed *p*-value of less than 0.05 was regarded as statistically significant.

## 3. Results and Discussion

Micronesian non-pregnancy-related hospitalizations accounted for 2.15% of the total sample (n = 3486) while white hospitalizations accounted for 44.3% (n = 71,827), Japanese hospitalizations accounted for 30.3% (n = 49,105), and Native Hawaiian hospitalizations accounted for 23.3% (n = 37,734). Briefly summarized, for all ethnicities about half of the samples were female and the majority were from Oahu. Specifically, 47.5% of Micronesians were female and 76.7% lived on Oahu. Eighty-two percent of the Micronesian sample was under 65, a significantly higher percentage than seen in other racial/ethnic groups; 65.7% of Native Hawaiians, 55.8% of whites and 27.1% of Japanese were under 65 (*p* < 0.001). Micronesians were significantly more likely than other studied groups to have Medicaid (*p* < 0.001) and self-pay health insurance (*p* < 0.001). Specifically, 75.2% of Micronesians had Medicaid, compared to 24.7% of Native Hawaiians, 15.6% of whites, and 3.3% of Japanese.

### 3.1. Total Costs

Table 1 shows the total costs by race/ethnicity of all non-pregnancy-related hospitalizations and the percent paid by Medicaid for each racial/ethnic group. The total cost of the 3486 hospitalizations of Micronesians during the study period was $58.1 million; 75% of this total was covered by Medicaid, which covered 23% of Native Hawaiian, 15% of white, and 3% of Japanese patient costs during the period.

**Figure 1:**
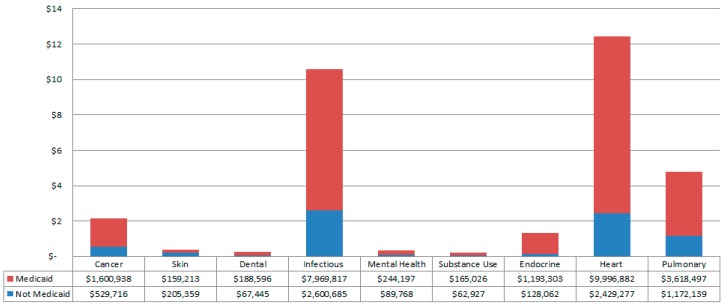
Total Costs (in Millions) for Micronesian hospitalizations by Medicaid *vs.* Not Medicaid by All Patient Refined Diagnosis Related Groups hospitalization types from 2010 to 2012 Hawaii Health Information Corporation Data. The amount paid by Medicaid for each type is noted. (n = 3486 Micronesian hospitalizations). Specific percentages as paid by Medicaid by disease type are as follows: 75% Cancer, 44% Skin, 74% Dental, 75% Infectious, 73% Mental Health, 72% Substance Use, 90% Endocrine, 80% Heart Disease, and 76% Pulmonary.

Figure 1 shows the total costs for Micronesians by type and by percent Medicaid payment. The two most expensive inpatient hospitalization types for Micronesians were cardiac-related (21% of total costs) and infectious disease-related (18% of total costs). For these, Medicaid paid 80% and 75% of total costs respectively.

**Table 1:** Totals Costs in US Dollars and in Percent Medicaid *vs.* Not Medicaid by Racial/Ethnic Group from all non-pregnancy related hospital discharges for Micronesians, Native Hawaiians, Japanese, and whites in Hawai‘i from 2010–2012 (n = 162,152 hospitalizations).

	Micronesian	Native Hawaiian	Japanese	White
Total n	3486	37,734	49,105	71,827
% of sample	2.2%	23.3%	30.3%	44.3%
Costs				
Total Costs	$58.1 million	$571.0 million	$725.1 million	$1051.0 million
Median Cost/hospitalization ^1^	$9833	$9349	$9752	$9595
Medicaid ^1^				
% Not Medicaid	25%	77%	97%	85%
% Medicaid	75%	23%	3%	15%

^1^ This varies significantly by race/ethnicity at *p* < 0.0001.

### 3.2. Unadjusted Costs

Table 1 also shows the median total cost by race/ethnicity. In unadjusted analyses, median costs varied significantly by race/ethnicity (*p* < 0.001). Micronesians had the highest median costs ($9993 per person) compared to $9752 for Japanese, $9594 for whites, and $9348 for Native Hawaiians.

**Figure 2:**
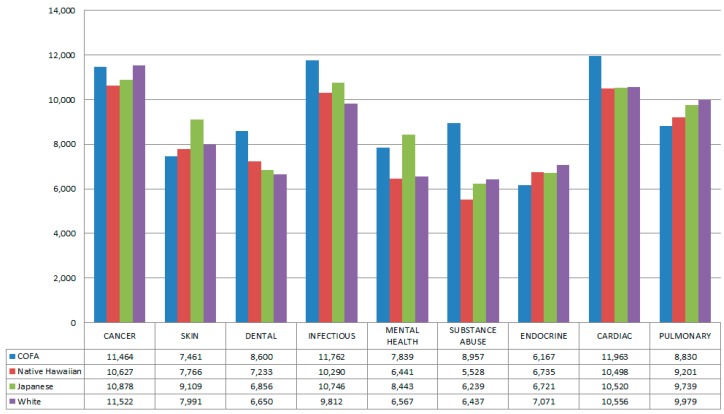
Median Costs in US Dollars by All Patient Refined Diagnosis Related Groups hospitalization-related types from 2010 to 2012 Hawaii Health Information Corporation Data. Cardiac, pulmonary, infectious, and mental health racial/ethnic comparisons were significant at *p* < 0.001. Substance abuse was significant at *p* < 0.01. (n = 69,449 unique individuals, at first visit). Comparisons by race were as follows, cancer (*p* = 0.15), endocrine (*p* = 0.07), cardiac (*p* < 0.001), pulmonary (*p* < 0.001), infectious (*p* < 0.001), mental health (*p* < 0.001), substance use (*p* = 0.004), skin (*p* = 0.36), and dental (*p* = 0.73).

Figure 2 illustrates the median, unadjusted costs overall and then by disease category types by race/ethnicity within each disease category.

In unadjusted analyses, Micronesians had the highest median hospitalization costs overall and for cardiac, infectious disease, and substance abuse-related hospitalizations. The substance abuse category includes all drug and alcohol abuse and dependence-related hospitalizations, including but not limited to opioids and cocaine. Significant differences (*p* < 0.05) were seen by race/ethnicity for all these categories. Micronesians also had the highest costs for dental hospitalizations, but the racial/ethnic comparison was not statistically significant for this disease category type. Costs for mental health-related hospitalizations also varied significantly by race/ethnicity (*p* < 0.001) and Micronesians had the second highest median costs for these hospitalizations (after Japanese).

Micronesians had the lowest median costs for the pulmonary disease category type, which differed significantly by race/ethnicity (*p* < 0.001). Micronesians also had the lowest median costs for endocrine and skin-related hospitalizations, but the racial/ethnic comparison was not significant for these disease category types. Median costs for cancer also did not differ significantly by race/ethnicity.

### 3.3. Multivariable Models

#### 3.3.1. Without SOI

As can be seen in Table 2, in multivariable models controlling for age, gender, payer, and location of residence, Micronesians had significantly higher costs overall and for both cardiac and infectious diseases compared to the other three racial/ethnic groups. They also had significantly higher costs for substance use compared to Native Hawaiians.

Appendix Table A2 provides the estimates for other variables in these models. Age was associated with costs, though the relationship varied in direction by disease type. Women, when significant, had lower costs. Not living on Oahu, when significant, was associated with higher costs. Non-private payers often had significantly higher costs with self-pay having the highest comparative costs in a number of models.

#### 3.3.2. With SOI

As also shown in Table 2, even when SOI was included in multivariable models, Micronesians still had significantly higher overall costs compared to Native Hawaiians. Once SOI was considered, Micronesians only had significantly higher costs compared to whites for cancer and compared to Native Hawaiians for cardiology. In no disease category did they have significantly lower costs in any adjusted model.

Appendix Table A3 provides the estimates for other variables in these models. Age was less consistently associated with costs across these models, likely because of its relationship with the SOI metric. Women, when significant, had lower costs. Not living on Oahu, when significant, was associated with higher costs. Non-private payers often had significantly higher costs with self-pay having the highest comparative costs in a number of models.

**Figure 3:**
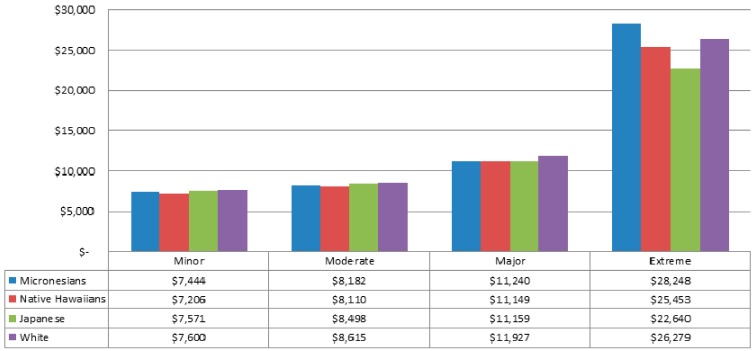
Median Costs in US Dollars by Race/Ethnicity and Severity of Illness Levels (Minor, Moderate, Major, Extreme) from 2010 to 2012 Hawaii Health Information Corporation Data. All four within-SOI-level racial/ethnic comparisons were significant at *p* < 0.001. (n = 162,152). Please note: The 3M Severity of Illness (SOI) score ranges from minor (1) to extreme (4) and is designed to be specifically used within APR-DRG disease categories. “Overall” SOI may thus not be a meaningful metric. Use of this overall metric here allows for a broad comparison of costs by SOI level for studying racial/ethnic groups, but these caveats should be considered.

Using the total sample (n = 162,152), Figure 3 compares median costs by race/ethnicity by the four SOI levels, minor, moderate, major, and extreme. As expected, for all racial/ethnic groups the median overall costs increased from minor to extreme SOI levels, with the largest difference between major and extreme SOI levels.

Within each SOI level, racial/ethnic comparisons were significant at *p* < 0.001, with the greatest differences seen within the extreme SOI level. Whites had the highest median costs for both minor and major SOI levels, Japanese had the highest median cost for the moderate SOI level, and Micronesians had the highest median cost in the extreme SOI level.

**Table 2:** Results of Multivariate Analysis for Costs with and without Severity of Illness (SOI) by race/ethnicity with Micronesians as the reference group. ^1^

		Costs (Not Including SOI)			Costs (Including SOI)		
		Native Hawaiian	Japanese	White	Native Hawaiian	Japanese	White
	n	Relative Risk (95% CI)	Relative Risk (95% CI)	Relative Risk (95% CI)	Relative Risk (95% CI)	Relative Risk (95% CI)	Relative Risk (95% CI)
Overall hospitalizations	162,152	0.88 (0.84–0.93)	0.82 (0.77–0.86)	0.83 (0.79–0.87)	0.95 (0.92–0.99)	0.97 (0.93–1.00)	0.99 (0.96–1.03)
Cancer	4172	1.16 (0.86–1.32)	1.07 (0.86–1.33)	1.11 (0.89–1.37)	1.04 (0.87–1.23)	1.12 (0.94–1.33)	1.15 (0.97–1.37)
Skin	997	0.95 (0.63–1.46)	0.94 (0.61–1.47)	0.92 (0.60–1.40)	1.11 (0.82–1.53)	1.12 (0.81–1.55)	1.12 (0.82–1.53)
Dental	694	0.88 (0.62–1.27)	0.84 (0.58–1.22)	0.81 (0.57–1.16)	0.85 (0.65–1.11)	0.81 (0.61–1.07)	0.84 (0.64–1.10)
Infectious	13,171	0.85 (0.74–0.98)	0.80 (0.69–0.93)	0.78 (0.67–0.90)	0.97 (0.88–1.06)	0.97 (0.88–1.07)	1.00 (0.91–1.10)
Mental Health	4993	0.89 (0.46–1.71)	1.06 (0.54–2.08)	0.81 (0.41–1.55)	0.86 (0.46–1.60)	1.01 (0.54–1.91)	0.81 (0.44–1.50)
Substance Abuse	1764	0.62 (0.39–0.999)	0.59 (0.36–0.97)	0.68 (0.43–1.08)	0.83 (0.59–1.18)	0.79 (0.55–1.14)	0.88 (0.62–1.23)
Endocrine	4055	1.05 (0.82–1.35)	1.00 (0.77–1.28)	0.99 (0.77–1.27)	1.10 (0.91–1.32)	1.11 (0.92–1.34)	1.11 (0.92–1.34)
Cardiac	26,127	0.86 (0.78–0.95)	0.85 (0.77–0.94)	0.83 (0.75–0.91)	0.88 (0.82–0.97)	0.94 (0.87–1.01)	0.93 (0.86–1.003)
Pulmonary	13,476	1.02 (0.88–1.17)	0.99 (0.86–1.14)	1.08 (0.94–1.24)	0.97 (.88–1.08)	1.03 (0.92–1.14)	1.08 (0.97–1.20)

^1^ All models include age, gender, payer, and location of residence. Other significant variables for the model without SOI can be found in Appendix Table A1. Other significant variables for the model with SOI can be found in Appendix Table A2.

### 3.4. Discussion

Micronesians had higher unadjusted hospitalization costs overall and specifically for several disease types compared to other racial/ethnic groups. In unadjusted analyses, Micronesians had the highest median hospitalization costs overall and for cardiac, infectious disease, and substance abuse-related hospitalizations. Importantly, infectious and heart disease were the two most expensive hospitalization types in term of total costs. In models adjusted for payer, age, gender, and location of residence, Micronesians still had significantly higher costs overall and for infectious and heart disease hospitalizations compared to all three other studied racial/ethnic groups.

Hospitalized Micronesians have higher SOI in many disease categories compared to Native Hawaiians, Japanese, and whites in Hawai‘i [24]. Thus, it is important to consider the role of SOI in explaining cost differences by race/ethnicity. The inclusion of severity of illness to the cost model explained some, but not all, of these higher costs. Even in final models, once SOI was controlled, Micronesians still had significantly higher overall costs compared to Native Hawaiians and had significantly higher cost for cardiac hospitalization compared to Native Hawaiians.

These findings may be of particular interests to state Medicaid programs. The Hawai‘i state Medicaid program was a major payer for Micronesian hospitalizations during this time period, particularly in some disease categories, including heart disease, where Medicaid was responsible for 80% of costs for Micronesian hospitalizations. As federal policies created the unique immigration status for this population while allowing them to be excluded from Medicaid participation under federal guidelines, a strong argument can be made that federal solutions are needed.

Our estimates suggests that one year of hospitals costs in Hawai‘i, if the size of the Micronesian population does not change and the case-mix does not change from the 2010–2012 years, is around $19 million total for public and private payers. For Medicaid alone, this estimate is $14.5 million. This figure accords with the cost cited in testimony by the Director of Human Services [15]. Inpatient costs are approximately 21% of annual health care costs [30].

This analysis sheds light on one important consequence of current policies to bar COFA migrants from Hawai‘i’s state Medicaid program: changes in the payer mix that will cover this population. Medicaid was covering 75% of overall costs for Micronesians, with higher percentages in some important disease categories—including cardiology (the highest studied cost category overall). While hospitals lose the reimbursements that they had received previously from Medicaid, coverage for COFA migrants aged 20 to 64 years previously on Medicaid has shifted to the two major Hawai‘i insurers participating in the Affordable Care Act (ACA) exchange: Kaiser and HMSA. As private insurance reimbursements are higher than those from Medicaid, hospitals may actually gain financially from this change.

Coverage plans available through the ACA are not designed for COFA migrants who may face significant linguistic, health literacy, and computer access challenges in enrollment. These plans may also not be appropriate for this population as the cost sharing under the private plans is substantially higher than under Medicaid. While the state is currently paying enrollment premiums for the COFA population moved from Medicaid, under the HMSA plan individuals are responsible for co-pays. (Kaiser is currently covering co-pays for this population.) Therefore Hawai‘i’s decision to move all non-ABD, non-pregnant, adult COFA migrants onto ACA insurance plans, even though they may fall below 133% of the federal poverty line, may decrease health care access for COFA migrants because they cannot afford the co-pays and any future premiums. Several surveys have found that co-pays and deductibles can deter people from seeking care in order to save money [31]. As noted by physician-anthropologist, Salmaan Keshavjee, “For the poor, ‘cost shifting’ often means no care at all.” ([32], p. 131)

The lack of attendance for primary and preventative care may result in patient’s presenting to emergency departments and being hospitalized with more severe and more costly conditions. This is especially relevant as a recent study found that Micronesians were already hospitalized younger and often sicker in a number of disease categories compared to Japanese, whites, and Native Hawaiians in Hawai‘i [24]. Thus, under new policies, the costs described here for Micronesian hospitalizations, which are already higher than for other racial/ethnic groups in the state, may rise.

These findings should be of interest not just to hospitals and insurance companies, but to many stakeholders, including advocates for health equity. While this research focused on the state of Hawai‘i, it has relevance for the other states with significant and/or growing populations of Micronesians, including Washington, Oregon, and Arkansas [1]. Additionally, many other states have populations who do not qualify for federal Medicaid services, but have high healthcare needs with little monetary resources [33]. These include many lawfully present immigrants who are subject to the 5-year waiting period for Medicaid and undocumented immigrants who are currently not eligible for most Medicaid services and who are, additionally, unable to purchase health coverage through state-level health insurance exchanges even at full price [33]. These non-citizens, who are as likely as citizens to be working, are less likely to have a usual source of preventive care and also to delay, or forgo, needed care due to cost [33]. While some states, like Hawai‘i, choose to use state funding to cover the costs of immigrant populations, in many cases these include very specific services or include only some groups (e.g., children and pregnant women), leaving others without coverage [33]. How to best provide all people residing within the US with comprehensive healthcare coverage, particularly given rising health care costs, will continue to be an important, and politically, divisive issue [12].

### 3.5. Limitations

There are several limitations to our study. Firstly the costs described here may not represent true costs as they are only an estimate. In addition, costs were determined from charges using a cost-to-charge ratio and do not represent necessarily the payments received for these services. Medicaid, for instance, often pays a significantly lower portion percentage of costs than Medicare or private payers. On the positive side, this means that our cost analysis is not confounded by the fact a wide variation was seen in the percent of hospitalizations by racial/ethnic group covered under Medicaid. In addition, the data included only non-pregnancy related hospitalizations of individuals over the age of 18 and therefore does not represent the total estimates of healthcare costs provided by the state to younger individuals, outpatient health care, and childbirth, which is the most common reason for hospitalization. We did not have data on individual hospitals so could not control for this in our models. Although we looked at overall costs, we did not analyze costs within all disease types. Instead we chose categories likely to be most relevant for Micronesians to help ensure we had samples large enough for reliable analyses. To be more comprehensive, we did our analyses by APR-DRG grouping rather than within each individual APR-DRG, despite the fact that the SOI measure is designed to be used only within individual APR-DRG categories. We did not include additional factors, such as length of stay or comorbidity in the multivariable models, which would have likely helped to explain cost differences by racial/ethnic groups. We also did not include a hospital variable in the multivariable models, which would have been useful. However, the cost adjustment factor is specific to each hospital. Although this analysis is not comprehensive, it does provide quantitative data to estimate the cost of inpatient health care for Micronesians overall and relative to other racial/ethnic groups. Finally, we compare costs, but it not clear whether higher cost signifies worse care or better care. The reasons for these differential costs by racial/ethnic group may be an important area for future research.

Diabetes is a good example of an area where future work on these topics, ideally with larger sample sizes, would be particularly relevant. Although diabetes is known to be a large problem for the Micronesian community, we did not find endocrine-related hospitalizations to be the largest overall total cost for this group, nor did Micronesians have a significantly higher median cost for this diagnostic grouping when compared to the other racial/ethnic groups. We would have liked to have considered diabetes-focused hospitalization specifically but the APR-DRG for diabetes-specific hospitalizations (420) included small numbers generally and very small numbers of Micronesians specifically. Additionally, the focus of our study was comparisons through the lenses of APR-DRGs, which codes hospitalizations based on the primary reason for each hospitalization. As diabetes is often comorbid with other conditions rather than the primary reasons for hospitalization, our methods would hide the full role of diabetes-related conditions on costs or outcomes. Future work considering diabetes-specific hospitalizations as well as the role of comorbid diabetes in Micronesian hospitalization costs and outcomes would be very useful. This would be particularly useful as there is evidence that Micronesians are hospitalized with endocrine-related diseases at significantly younger ages than the other three comparison racial/ethnic populations and significantly sicker than the Japanese [25]. These younger ages are not only significant as a marker for an increase in human suffering and potential of life-threatening issues at younger ages, but also potentially greater overall lifetime costs. These are important clinical and policy-related concerns to consider and better understand.

Similarly, another important issue to consider in future research is the link between infectious disease and heart disease, especially given that Pacific Islanders have a high prevalence of rheumatic heart disease [34]. This was not considered in this paper due to the focus on APR-DRGs, which assign only one primary diagnostic category per hospitalization. Future consideration of comorbidities around these topics might help to illuminate relevant health disparities or their consequences. As these two disease categories accounted for a large percentage of the total overall costs for the Micronesian population in Hawaii, these are very policy-relevant issues.

## 4. Conclusions

Micronesians have higher unadjusted hospitalization costs overall and specifically for several disease types. Higher SOI in Micronesians explains some, but not all of these higher costs. It is important to find the root causes of these apparent health inequities so that we may not only lower healthcare costs but decrease suffering. Based on our findings, we suggest health equity research should consider the root causes of the higher SOI in Micronesians to address these high healthcare cost burdens. Policy makers should include Micronesians as a covered group under federal Medicaid guidelines to provide an important infrastructure to sustain coverage of this population and improve their population health outcomes.

## Appendix

**Table A1:** Detail about APR-DRG-defined Disease Categories based on groupings of 3M’s APR-DRGs.

Disease Category	Sub-Category	Specific APR-DRG Codes Included
Cancer	Cancer	003, 041, 110, 136, 240, 281, 343, 362, 382, 442, 461, 500, 511, 512, 530, 690, 691, 693, 694
Chronic Disease	Endocrine	420, 421, 422, 423, 424, 425
	Heart	022, 045, 047, 160, 161, 162, 163, 165, 166, 167, 170, 171, 173, 174, 175, 176, 177, 180, 190, 191, 192, 193, 194, 196, 197, 198, 199, 200, 201, 203, 204, 205, 206, 207
	Pulmonary	113, 120, 121, 130, 131, 132, 133, 134, 135, 137, 138, 139, 140, 141, 142, 143, 144
Infectious	Infectious	049, 051, 050, 344, 383, 710, 720, 723, 724, 890, 892, 893, 894
Behavioral	Mental Health	740, 750, 751, 752, 753, 754, 755, 756, 757, 758, 759, 760
	Substance Abuse	280, 770, 772, 773, 774, 775, 776
Other	Skin	380, 381, 384, 385
	Dental	098, 114, 115

**Table A2:** Significant Variables in Multivariable Models without Severity of Illness (SOI).

	Cancer	Skin	Dental	Infectious	Mental Health	Substance Abuse	Endocrine	Cardiac	Pulmonary	Overall
	Coefficient (95% CI)	Coefficient (95% CI)	Coefficient (95% CI)	Coefficient (95% CI)	Coefficient (95% CI)	Coefficient (95% CI)	Coefficient (95% CI)	Coefficient (95% CI)	Coefficient (95% CI)	Coefficient (95% CI)
**Age**	0.999 (0.996–1.002)	1.001 (1.00–1.01)	1.01 (1.004–1.01)	1.001 (1.000–1.003)	1.01 (1.01–1.02)	1.018 (1.015–1.022)	1.001 (0.998–1.004)	0.997 (0.996–0.998)	1.000 (0.998–1.001)	1.003 (1.0028–1.004)
**Female**	0.85 (0.80–0.91)	1.02 (0.88–1.18)	0.99 (0.87–1.12)	1.91 (0.87–0.96)	1.04 (0.94–1.15)	0.93 (0.84–1.02)	0.97 (0.90–1.05)	0.89 (0.86–0.91)	0.94 (0.90–0.97)	0.89 (0.87–0.90)
**Location**										
Hawai‘i, not Oahu	1.04 (0.97–1.11)	1.02 (0.87–1.19)	1.09 (0.95–1.24)	1.04 (0.98–1.10)	1.40 (1.26–1.54)	1.21 (1.09–1.33)	1.14 (1.04–1.25)	1.09 (1.06–1.13)	1.06 (1.02–1.11)	1.05 (1.03–1.07)
Oahu	-	-	-	-	-	-	-	-	-	-
Not from Hawai‘i	0.90 (0.75–1.07)	0.71 (0.51–0.99)	0.89 (0.68–1.15)	0.98 (0.88–1.11)	1.17 (0.90–1.51)	1.42 (1.14–1.78)	1.04 (0.85–1.27)	1.08 (1.02–1.14)	0.96 (0.89–1.04)	1.02 (0.98–1.05)
**Payer**										
DOD	0.92 (0.69–1.22)	1.14 (0.67–1.95)	1.23 (0.83–1.84)	1.09 (0.90–1.33)	0.92 (0.69–1.21)	1.62 (1.20–2.20)	1.42 (0.99–2.03)	1.03 (0.93–1.13)	1.05 (0.91–1.20)	1.07 (1.02–1.13)
Medicaid	1.17 (1.04–1.30)	1.17 (0.92–1.49)	1.10 (0.90–1.32)	1.01 (0.93–1.09)	1.02 (0.90–1.15)	1.05 (0.93–1.18)	1.08 (0.93–1.25)	1.04 (0.99–1.10)	1.02 (0.95–1.10)	1.03 (1.00–1.05)
Medicare	1.15 (1.06–1.26)	1.02 (0.88–1.36)	0.89 (0.75–1.07)	1.10 (1.03–1.18)	1.34 (1.15–1.56)	1.00 (0.86–1.17)	1.05 (0.93–1.18)	1.09 (1.05–1.13)	1.11 (1.05–1.18)	1.07 (1.05–1.10)
Private	-	-	-	-	-	-	-	-	-	-
Self-Pay	1.41 (1.08–1.85)	1.02 (0.76–1.37)	1.26 (0.98–1.61)	0.92 (0.81–1.06)	0.88 (0.72–1.08)	0.93 (0.79–1.10)	0.92 (0.69–1.23)	1.03 (0.94–1.12)	0.91 (0.81–1.03)	1.14 (1.10–1.19)

“-” Means reference group.

**Table A3:** Significant Variables in Multivariable Models with Severity of Illness (SOI).

	Cancer	Skin	Dental	Infectious	Mental Health	Substance Abuse	Endocrine	Cardiac	Pulmonary	Overall
	Coefficient (95% CI)	Coefficient (95% CI)	Coefficient (95% CI)	Coefficient (95% CI)	Coefficient (95% CI)	Coefficient (95% CI)	Coefficient (95% CI)	Coefficient (95% CI)	Coefficient (95% CI)	Coefficient (95% CI)
**Severity of Illness**	1.50 (1.46–1.54)	1.60 (1.50–1.71)	1.46 (1.38–1.54)	1.87 (1.84–1.90)	1.38 (1.28–1.49)	1.68 (1.61–1.75)	1.51 (1.46–1.56)	1.46 (1.45–1.48)	1.58 (1.55–1.60)	1.56 (1.55–1.56)
**Age**	0.99 (0.992–0.991)	1.004 (0.999–1.007)	1.00 (1.00–1.01)	0.995 (0.993–0.996	1.01 (1.004–1.01)	1.01 (1.0003–1.009)	1.00 (0.998–1.003)	0.99 (0.99–0.995)	0.9967 (0.9956–0.998)	0.9995 (0.998–0.9999)
**Female**	0.98 (0.93–1.03)	1.05 (0.95–1.53)	1.02 (0.93–1.12)	0.96 (0.93–0.99)	1.03 (0.94–1.13)	0.98 (0.91–1.06)	1.01 (0.95–1.07)	0.88 (0.86–0.90)	0.99 (0.96–1.02)	0.95 (0.94–0.95)
**Location**										
Hawai‘i, not Oahu	1.04 (0.98–1.09)	1.10 (0.98–1.23)	1.11 (1.01–1.23)	1.16 (1.12–1.21)	1.41 (1.28–1.55)	1.20 (1.12–1.29)	1.17 (1.10–1.25)	1.12 (1.10–1.15)	1.13 (1.09–1.17)	1.12 (1.10–1.13)
Oahu	-	-	-	-	-	-	-	-	-	-
Not from Hawai‘i	0.80 (0.70–0.93)	0.91 (0.82–1.53)	0.93 (0.76–1.14)	1.05 (0.96–1.13)	1.18 (0.93–1.51)	1.31 (1.12–1.54)	1.12 (0.97–1.30)	1.09 (1.05–1.14)	1.00 (0.94–1.07)	1.03 (1.01–1.06)
**Payer**										
DOD	0.86 (0.69–1.07)	1.12 (0.95–1.17)	1.19 (0.88–1.62)	1.09 (0.96–1.24)	0.91 (0.70–1.18)	1.33 (1.06–1.66)	1.24 (0.95–1.14)	0.92 (0.85–1.00)	1.02 (0.92–1.12)	0.97 (0.93–1.00)
Medicaid	1.00 (0.91–1.09)	1.17 (0.98–1.40)	1.02 (0.88–1.18)	1.07 (1.01–1.12)	1.01 (0.89–1.13)	1.06 (0.97–1.15)	1.03 (0.92–1.14)	0.95 (0.91–0.99)	0.99 (0.93–1.04)	0.93 (0.92–0.95)
Medicare	1.05 (0.97–1.12)	0.96 (0.81–1.13)	0.89 (0.77–1.01)	1.04 (0.99–1.09)	1.27 (1.10–1.46)	1.07 (0.96–1.20)	0.98 (0.90–1.08)	1.00 (0.97–1.03)	1.04 (0.99–1.08)	0.97 (0.95–0.98)
Private	-	-	-	-	-	-	-	-	-	-
Self-Pay	1.12 (0.91–1.39)	1.17 (0.94–1.46)	1.17 (0.98–1.41)	1.08 (0.99–1.19)	0.90 (0.74–1.09)	0.97 (0.86–1.10)	0.92 (0.74–1.14)	0.92 (0.86–0.99)	0.95 (0.87–1.04)	1.05 (1.02–1.08)

“-” Means reference group.
